# Stage-Specific Effects of Silver Nanoparticles on Physiology During the Early Growth Stages of Rice

**DOI:** 10.3390/plants13233454

**Published:** 2024-12-09

**Authors:** Ruxue Pan, Zailin Zhang, Ya Li, Sihong Zhu, Sumera Anwar, Jiaquan Huang, Chuanling Zhang, Liyan Yin

**Affiliations:** 1 School of Life and Health Sciences, Hainan Province Key Laboratory of One Health, Collaborative Innovation Center of One Health, Hainan University, Haikou 570228, China; rux_pan@163.com (R.P.); 17889897053@163.com (Z.Z.); 18489075962@163.com (Y.L.); 13190611305@163.com (S.Z.); 2 Department of Botany, Government College Women University Faisalabad, Faisalabad 38000, Pakistan; sumeraanwar@mail.hzau.edu.cn; 3 School of Breeding and Multiplication, Sanya Institute of Breeding and Multiplication, Hainan University, Sanya 572022, China; jqhuang@hainanu.edu.cn

**Keywords:** AgNPs, rice, growth stage, antioxidant system, photosynthetic system

## Abstract

Silver nanoparticles (AgNPs), widely utilized nanomaterials, can negatively affect crop growth and development. However, it remains unclear whether crops exhibit similar responses to AgNPs stress at seed germination and seedling stages. In this study, rice seeds and seedlings were exposed to AgNPs, and their growth, photosynthetic efficiency, and antioxidant systems were recorded. demonstrated significant AgNPs accumulation in rice tissues, with notable higher accumulation in seedlings exposed to AgNPs after germination compared to AgNPs exposure during germination. The roots exhibited greater AgNPs accumulation than shoots across both stages. Exposure to AgNPs during the seed germination stage, even at concentrations up to 2 mg/L, did not significantly affect growth, physiological indices, or oxidative stress. In contrast, seedlings exposed to 1 and 2 mg/L AgNPs showed significant reductions in shoot length, biomass, nutrient content, and photosynthetic efficiency. At low AgNPs concentrations, the maximum relative electron transport rate (rETR_max_) was significantly reduced, while the higher concentrations caused pronounced declines in the chlorophyll a fluorescence transient curves (OJIP) compared to the control group. Antioxidant enzyme activities increased in both leaves and roots in a dose-dependent manner, with roots exhibiting significantly higher activity, suggesting that roots are the primary site of AgNPs stress responses. In conclusion, rice responds differently to AgNPs exposure at distinct developmental stages, with the seedling stage being more susceptible to AgNPs-induced stress than the seed germination stage. These findings underscore the importance of considering growth stages when assessing the food safety and environmental risks associated with AgNPs exposure.

## 1. Introduction

In recent years, the development of nanotechnology has led to widespread applications of engineered nanomaterials in various fields [1]. Among these materials, silver nanoparticles (AgNPs) have increased due to their broad-spectrum antibacterial and plasmonic properties [2]. AgNPs are commonly found in various commercial products, including textiles, cosmetics, home appliances, and medical devices [3,4]. Research indicates that nearly 450 products incorporate AgNPs, making them the most produced nanoparticle among all nanomaterials. The widespread use of AgNPs-containing products results in their release into the environment [5], with a significant portion ending up in wastewater treatment plants, landfills, and surface waters [6]. The application of wastewater irrigation and sludge contributes to the entry of AgNPs into agricultural ecosystems [7]. Presently, AgNPs concentrations in agricultural soils range from 0.24 to 792.23 ng/kg, with projections suggesting that 2050 levels could reach up to 10 µg/kg [8]. AgNPs are utilized as nano-fungicides for direct application on leaf surfaces to combat pathogens like *Pyricularia* sp. [9], *Botrytis cinerea* [10], and *Fusarium graminearum* [11]. Both root irrigation with AgNPs-containing wastewater/sludge and foliar application of AgNPs as pesticides significantly heighten the risk of direct contact between AgNPs and crops [12,13].

Rice (*Oryza sativa* L.) is an important staple food for half of the world’s population [14]. In 2019, the global rice cultivation area reached 167 million hectares, ranking it third in production [15]. Rice cultivation primarily employs direct seeding and transplanting methods. Direct seeding involves sowing seeds directly into the field for germination and root establishment, which can significantly reduce labor requirements [16]. The transplanting method entails planting rice seedlings in the field, which can lower pest and disease incidence and increase yields [17]. Different field planting methods lead to varying initial growth stages of rice, allowing rice at seed germination and seedling stages to contact and absorb AgNPs in natural agricultural settings directly. As rice grows, the AgNPs absorbed and accumulated in the rice may move through the food chain, potentially posing significant threats to food safety and human health [5,18]. With the increasing release of AgNPs into agricultural ecosystems, researchers are increasingly focusing on the impact of AgNPs on crop growth and development [19].

Evidence suggests that AgNPs have negatively affected the physiological level of plants [20,21]. For instance, AgNPs can inhibit plant growth and development, affecting the grain quality of crops [22,23]. *Triticum aestivum* L. cultivated in soil with 2000 mg/kg AgNPs exhibits notable reductions in biomass, plant height, microelement content, arginine, and histidine in grains, affecting grain quality [22]. Rice seedlings exposed to 100 and 1000 mg/L AgNPs solutions show significant decreases in root fresh weight, dry weight, and root length [23]. 

AgNPs can also have adverse effects on plant photosynthesis. Previous studies have demonstrated that AgNPs can cause significant damage to the chloroplast structure in the leaves of *Hordeum vulgare* L. [24]. This disruption leads to a reduction in the content of chlorophyll and carotenoids, which are crucial pigments for photosynthesis. Additionally, there is a decline in key chlorophyll a fluorescence parameters such as the maximal quantum yield (F_v_/F_m_) and nonphotochemical quenching (NPQ), indicating compromised photosynthetic efficiency [24,25,26]. Moreover, in *Oryza sativa* L., exposure to AgNPs can disturb the delicate balance of redox homeostasis. This imbalance results in the overproduction of reactive oxygen species (ROS), which are highly reactive molecules that can inflict oxidative damage on vital cellular components, including lipids, proteins, and DNA [25,27]. To counteract this oxidative stress, the activity of various antioxidant enzymes is activated, although this response may not fully mitigate the damage.

The interaction between AgNPs and plant systems can trigger a cascade of metabolic and proteomic responses [28]. For instance, a study conducted by Jhanzab et al. [29] employed proteomic analysis on wheat plants exposed to AgNPs. Their findings revealed an intriguing pattern: proteins associated with photosynthesis and protein synthesis were found to be significantly elevated. In contrast, proteins linked to glycolysis, signaling pathways, and cell wall integrity exhibited a marked decrease. Additionally, there was a reduction in the levels of proteins involved in redox processes and the mitochondrial electron transport chain, highlighting the profound impact of AgNPs exposure on plant metabolism.

The phytotoxic effects of AgNPs are influenced by various factors, including concentration, particle size, shape, solubility, and coating materials [30]. Research indicates that particle size significantly impacts the toxicity of AgNPs, with smaller particles generally demonstrating higher toxicity [31]. For instance, compared to 60 nm AgNPs, 10 nm AgNPs lead to increased silver accumulation and toxicity in the roots of *Lactuca sativa* [32]. Exposure of *Lactuca sativa* roots to different concentrations of AgNPs (0.1, 0.5, 1 mg/L) results in a dose-dependent decrease in both root and shoot biomass with increasing AgNPs concentration [33]. Furthermore, the coatings on AgNPs affect plants’ absorption. Studies have shown that after 7 days of treatment with 100 μM AgNPs, the silver content in *Nicotiana tabacum* L. was higher in CTAB-AgNPs (56 nm) treatment (8.84 μg/g) compared to citrate-AgNPs (24 nm) treatment (3.65 μg/g) [34]. Different growth stages of plants respond diversely to environmental pollution [35], but it remains unclear whether the toxic effects of AgNPs are linked to the plant growth stage. Most existing research has focused on treating plants at a single growth stage with AgNPs [33,36,37,38,39], lacking exploration of the differences in AgNPs’ toxicity effects across various growth stages under consistent experimental conditions. In summary, current research has overlooked the possibility that rice at different growth stages may be directly exposed to AgNPs in natural ecological environments. Therefore, it can be reasonably assumed that AgNPs exposure during germination and after germination will have varying effects on subsequent growth, leading to varying physiological responses. Studies concentrating on toxicity effects at a single growth stage may not comprehensively evaluate the environmental impact of AgNPs. Therefore, this study employs different concentrations of AgNPs to treat rice seeds and seedlings at seed germination and seedling stages to examine the variations in AgNPs’ effects on rice growth, development, photosynthesis, and antioxidant systems. The objective is to establish a theoretical foundation for assessing the food safety and environmental toxicity effects of AgNPs exposure at different plant growth stages.

## 2. Results

### 2.1. Ag Accumulation Measurement in Rice Tissues

**Figure 1:**
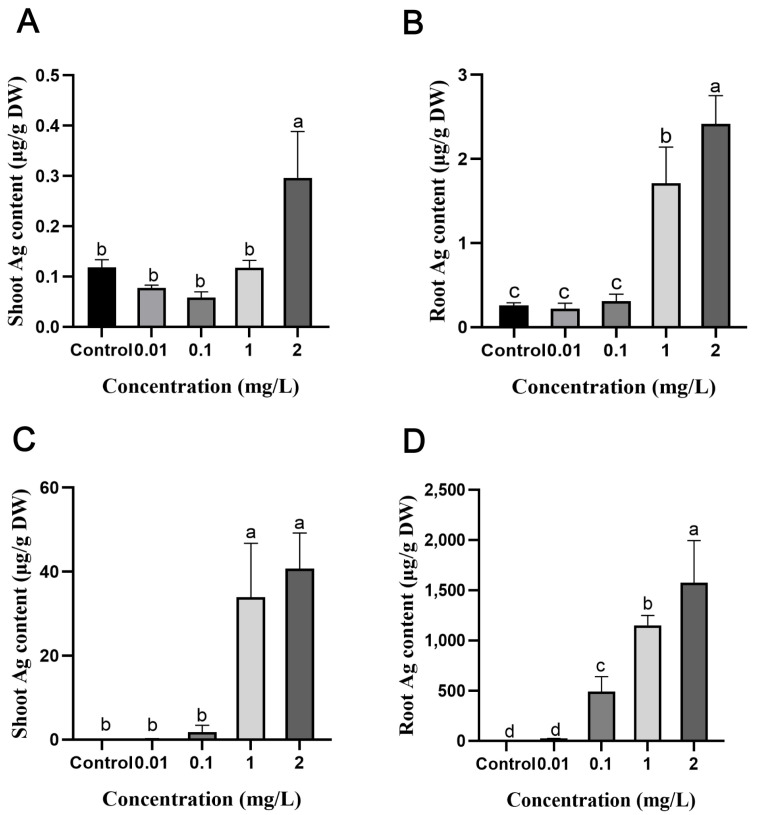
Ag accumulation in rice tissues exposed to AgNPs at the seed germination and seedling stages. AgNPs exposure at the seed germination stage: (**A**) Ag content in the shoots and (**B**) Ag content in the roots. For AgNPs exposure at the seedling stage: (**C**) Ag content in the shoots and (**D**) Ag content in the roots. Data are presented as mean ± SD. According to Tukey’s test (*p* < 0.05), different lowercase letters indicate significant differences among treatments (*n* = 3).

The rice seeds were exposed to AgNPs for 5 days during seed germination, and half of the seedlings were harvested to determine Ag accumulation in different tissues, including the endosperm, coleoptile, and root. The results showed that the lowest accumulation was observed in the endosperms, followed by the coleoptile, and the highest was observed in the radicle (Figure S1A). At 1 mg/L and 2 mg/L AgNPs exposure, the Ag content in the coleoptiles reached 5.6 µg/g dry weight (DW) and 13.2 µg/g DW, respectively, while in the radicles, it was significantly higher at 61.1 µg/g DW and 118.9 µg/g DW, respectively (Figure S1B,C). The rest of the seedlings exposed to AgNPs during germination were harvested after 13 days of normal growth. Ag content was then measured in the shoots and roots of seedlings (Figure 1). The Ag content in the shoots was reduced to 0.12 µg/g DW and 0.30 µg/g DW, respectively, and in the roots it was reduced to 1.7 µg/g DW and 2.4 µg/g DW, respectively (Figure 1A,B). 

When rice seedlings were exposed to AgNPs, the Ag content accumulated in the shoots was 33.9 µg/g DW and 40.7 µg/g DW at 1 and 2 mg/L AgNPs, respectively (Figure 1C). Whereas in the roots, it was 1154.1 µg/g DW and 1575.4 µg/g DW, respectively, which corresponds to 34 times and 39 times the Ag accumulation in the shoots (Figure 1D). In summary, Ag accumulation in rice is concentration- and developmental-stage dependent. Seedlings accumulated considerably more Ag than seeds during germination, especially at higher AgNPs concentrations. Additionally, regardless of the developmental stage, the rice roots consistently accumulated more Ag than the shoots.

### 2.2. Effects of AgNPs on Rice Seedling Growth

**Figure 2:**
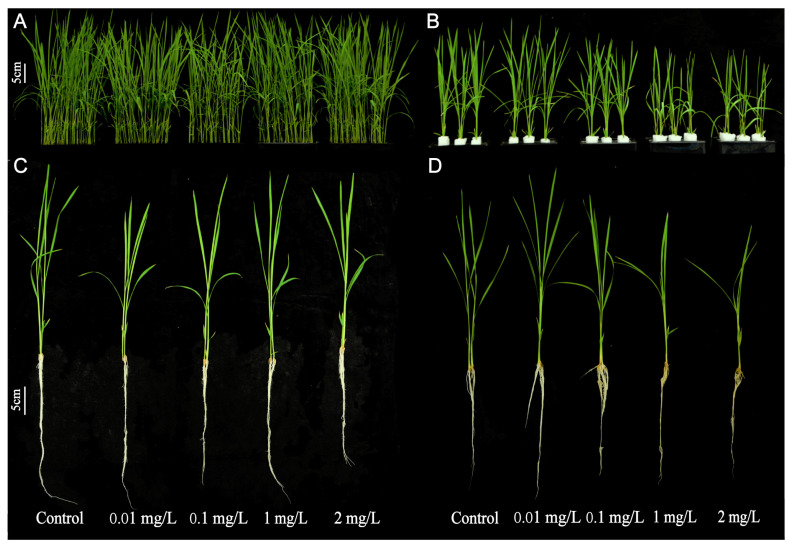
Growth phenotypes of rice seedlings exposed to AgNPs at seed germination and seedling stages. AgNPs exposure at the seed germination stage: (**A**,**C**). AgNPs exposure at the seedling stage: (**B**,**D**).

**Figure 3:**
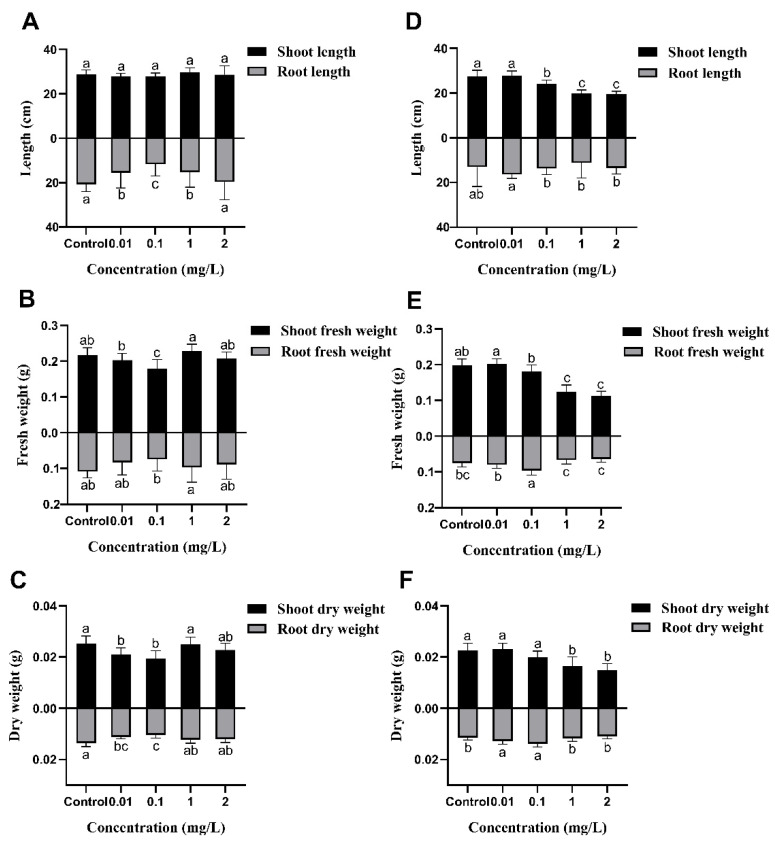
Effects of AgNPs exposure at seed germination and the seedling stages on rice growth parameters. AgNPs exposure at the seed germination stage: (**A**) shoot length and root length, (**B**) shoot fresh weight and root fresh weight, and (**C**) shoot dry weight and root dry weight. AgNPs exposure at the seedling stage: (**D**) shoot length and root length, (**E**) shoot fresh weight and root fresh weight, and (**F**) shoot dry weight and root dry weight. Data are presented as mean ± SD. According to Tukey’s test (*p* < 0.05), different lowercase letters indicate significant differences among treatments, *n* ≥ 4.

The growth phenotype of rice seedlings is shown in Figure 2. Compared to the control, AgNPs exposure during seed germination did not significantly affect the subsequent growth of rice seedlings (Figure 2A,C). The statistical analysis indicates that 1 and 2 mg/L AgNPs exposure during seed germination had no significant effect on the shoot length, shoot fresh weight, shoot dry weight, root dry weight, total fresh weight, or total dry weight of rice seedlings compared to the control (Figure 3 and Figure S2). However, exposure to 0.1 mg/L AgNPs significantly reduced the root length, shoot fresh weight, shoot dry weight, and root dry weight of rice seedlings by 35.3%, 18.0%, 23.1%, and 23.4%, respectively, to the control (Figure 3). Additionally, total fresh weight and total dry weight decreased by 19.3% and 23.2%, respectively (Figure S2).

In contrast, exposure to AgNPs at the seedling stage significantly inhibited the growth of rice seedlings. As AgNPs concentration increased, the height of the rice seedlings was noticeably reduced. At higher concentrations of 1 and 2 mg/L AgNPs exposure, the roots of rice seedlings were severely damaged and exhibited yellowing (Figure 2B,D). Increasing the AgNPs concentration also resulted in a pronounced reduction in shoot length (Figure 3D), shoot fresh weight (Figure 3E), and shoot dry weight (Figure 3F). Root length, however, did not show a significant decrease compared to the control. At 1 and 2 mg/L AgNPs, the shoot length decreased by 27.0% and 28.0%, the shoot fresh weight decreased by 37.1% and 42.7%, the root fresh weight decreased by 11.0% and 15.1% (Figure 3), and the total fresh weight decreased by 29.9% and 35.1%, respectively, as compared to the control (Figure S2).

In summary, the effects of AgNPs exposure on rice seedling growth differed based on the developmental stage of exposure. High concentrations of AgNPs during the seed germination stage had no significant effect on shoot and root growth at later stages. However, exposure to high concentrations of AgNPs during the seedling stage significantly inhibited growth and reduced biomass in rice seedlings.

### 2.3. Effects of AgNPs on Rice Photosynthesis

**Figure 4:**
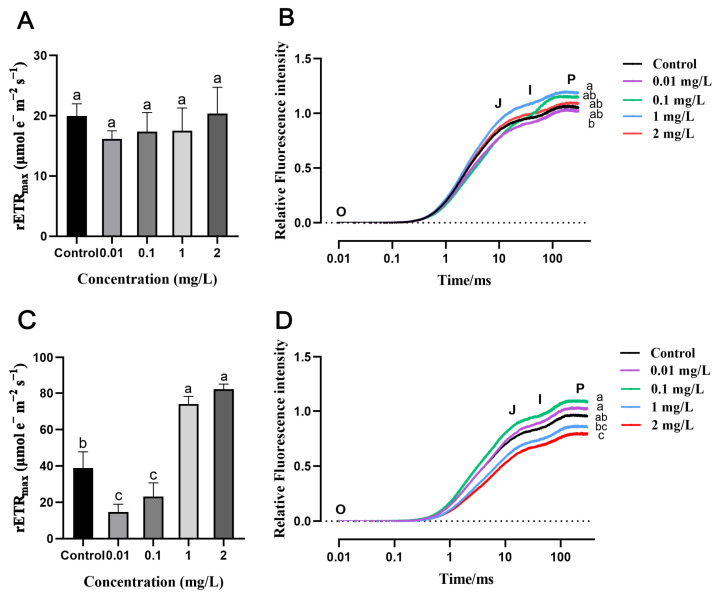
Effects of AgNPs exposure at seed germination and seedling stages on leaf photosynthetic parameters. AgNPs exposure at the seed germination stage: (**A**) maximum relative electron transport rate (rETR_max_) and (**B**) chlorophyll a fluorescence transient curves (OJIP). AgNPs exposure at the seedling stage: (**C**) rETR_max_ and (**D**) OJIP curves. Data are presented as mean ± SD. According to Tukey’s test (*p* < 0.05), different lowercase letters indicate significant differences among treatments, *n* ≥ 4.

Chlorophyll fluorescence measurement results showed that exposure to AgNPs at the seed germination stage does not significantly differ in F_v_/F_m_ and the maximum relative electron transport rate (rETR_max_) of rice seedlings compared to the control (Figure S5A and Figure 4A). However, exposure to 0.01 and 0.1 mg/L AgNPs during the seedling stage, rETR_max_, was significantly decreased by 62.1% and 40.3%, respectively (Figure 4C). In contrast, 1 and 2 mg/L AgNPs increased rETR_max_ by 91.0% and 112.1%, respectively (Figure 4C). In addition, the chlorophyll a fluorescence transient curves (OJIP) of rice seedlings were not significantly affected by AgNPs at the seed germination stage but were impacted at the seedling stage (Figure 4B,D). The OJIP fluorescence transient curves of 0.01 and 0.1 mg/L AgNPs treatment groups showed slightly higher values compared to the control group but with no significant difference. As the treatment concentrations increased, the OJIP fluorescence transient curves began to decline, with 1 and 2 mg/L AgNPs treatment groups significantly lower than those of the control (Figure 4D). In summary, the effects of AgNPs on the photosynthesis of rice seedlings vary depending on the growth stage at which the exposure occurs. 

### 2.4. Effects of AgNPs on Hydrogen Peroxide (H_2_O_2_), ROS, and Malondialdehyde (MDA) Contents in Rice Leaves

**Figure 5:**
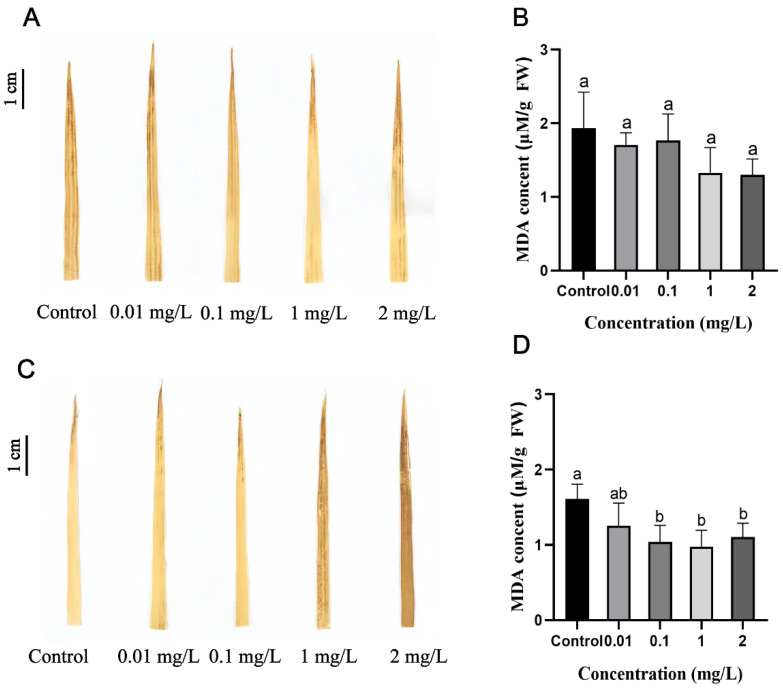
Effects of AgNPs exposure at seed germination and seedling stages on antioxidant substances in rice leaves. AgNPs exposure at the seed germination stage: (**A**) 3,3′-diaminobenzidine (DAB) staining image and (**B**) malondialdehyde (MDA) content. AgNPs exposure at the seedling stage: (**C**) DAB staining image and (**D**) MDA content. The MDA data are presented as mean ± SD. According to Tukey’s test (*p* < 0.05), different lowercase letters indicate significant differences among treatments, *n* ≥ 3.

The exposure of AgNPs during the seed germination stage did not result in significant differences in H_2_O_2_ and MDA contents in the leaves of the rice seedlings during subsequent growth compared to the control (Figure 5A,B). Still, it reduced ROS levels (Figure S6A). During the seedling stage, as the concentration of AgNPs increased, the brown coloration of the rice leaves deepened, indicating a gradual increase in H_2_O_2_ content, with the highest H_2_O_2_ content observed at 2 mg/L AgNPs (Figure 5C). However, the MDA content decreased as the AgNPs concentration increased (Figure 5D). Additionally, under low concentrations (0.01 and 0.1 mg/L) of AgNPs exposure, the ROS content in the leaves was significantly lower than in the control. At the same time, there was no significant difference in ROS content between the high concentrations of AgNPs (1 and 2 mg/L) and the control (Figure S6B). The above results indicate that AgNPs exposure at seed germination and seedling stages affects the oxidative damage of rice seedling leaves differently at a later growth. Exposure to AgNPs at the seed germination stage resulted in less or no oxidative damage to rice leaves than exposure at the seedling stage.

### 2.5. Effects of AgNPs on ROS and MDA Contents in Rice Roots

**Figure 6:**
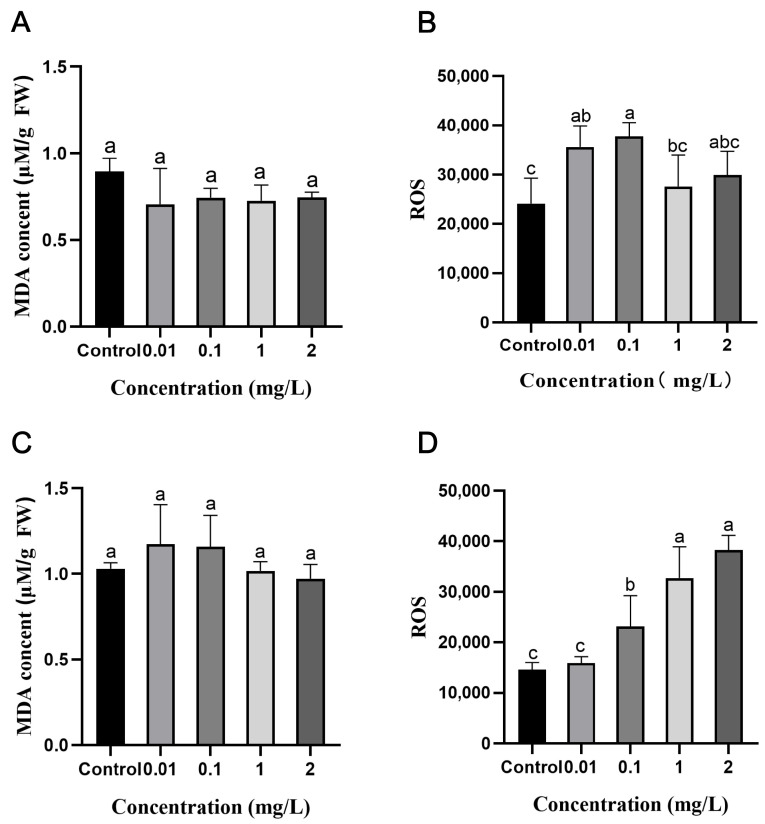
Effects of AgNPs exposure at seed germination and seedling stages on antioxidant substances in rice roots. AgNPs exposure at the seed germination stage: (**A**) MDA content and (**B**) ROS fluorescence intensity. AgNPs exposure at the seedling stage: (**C**) MDA content and (**D**) ROS fluorescence intensity. Data are presented as mean ± SD. According to Tukey’s test (*p* < 0.05), different lowercase letters indicate significant differences among treatments, *n* ≥ 3.

Exposure of AgNPs during the seed germination stage resulted in a lower, though not significantly different, MDA content in the rice roots compared to the control (Figure 6A). Under low concentrations of AgNPs (0.01 and 0.1 mg/L), ROS content significantly increased, while there was no significant difference at high AgNPs concentrations (1 and 2 mg/L) compared to the control (Figure 6B). Seedlings exposure to AgNPs resulted in MDA content in the rice roots initially increasing and then decreasing with higher AgNPs concentrations. However, no significant differences were observed compared to the control (Figure 6C). The ROS content in the rice roots gradually increased with increasing AgNPs concentration and significantly increased by 124% and 162% at high concentrations (1 and 2 mg/L), respectively (Figure 6D).

The results indicate that exposure to AgNPs at seed germination and seedling stages leads to varying levels of oxidative damage to rice roots. Specifically, AgNPs exposure at the seedling stage caused a significantly greater increase in ROS content in the roots than at the seed germination stage. However, no significant differences in MDA content were observed in the roots between AgNPs exposure groups and the control group at either growth stage.

### 2.6. Effects of AgNPs on Protein Content and Antioxidant Enzyme Activity in Rice Leaves

**Figure 7:**
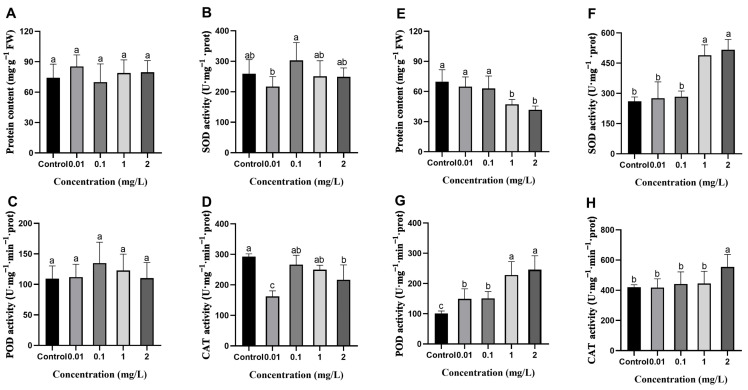
Effects of AgNPs exposure at seed germination and seedling stages on antioxidant enzyme activities in rice leaves. AgNPs exposure at the seed germination stage: (**A**) protein content, (**B**) SOD activity, (**C**) POD activity, and (**D**) CAT activity. AgNPs exposure at the seedling stage: (**E**) protein content, (**F**) SOD activity, (**G**) POD activity, and (**H**) CAT activity. Data are mean ± SD. According to Tukey’s test (*p* < 0.05), different lowercase letters indicate significant differences among treatments, *n* ≥ 3.

According to Figure 5 and Figure 6, rice tissues produce ROS under AgNPs exposure. The antioxidant system, including antioxidant enzymes and antioxidant substances, is used to remove oxidative stress substances and maintain homeostasis [40]. The protein content in the rice seedling leaves after exposure to AgNPs during the seed germination stage showed no significant difference compared to the control (Figure 7A). The AgNPs exposure at the seedling stage resulted in a progressive decrease in protein content in the rice seedling leaves with increasing AgNPs concentration. Low concentrations of AgNPs (0.01 and 0.1 mg/L) did not significantly impact protein content, while high concentrations (1 and 2 mg/L) resulted in a significant reduction of 32.4% and 40.3%, respectively, compared to the control (Figure 7E). 

Antioxidant enzymes mainly include superoxide dismutase (SOD), peroxidase (POD), catalase (CAT), and ascorbate peroxidase (APX), which can scavenge excessive ROS to maintain cellular homeostasis. Following exposure to AgNPs at the seed germination stage, the SOD and POD activities in the rice seedling leaves showed no significant differences compared to the control (Figure 7B,C). CAT activity was reduced relative to the control. The lowest reduction was observed at 0.01 mg/L, showing a 44.5% decrease compared to the control (Figure 7D). However, under AgNPs exposure at the seedling stage, SOD, POD, and CAT activities in rice seedling leaves were higher than those in the control and increased gradually with higher AgNPs concentrations. Upon exposure to high concentrations of AgNPs (1 and 2 mg/L) at the seedling stage, compared to the control, SOD activity increased by 87.7% and 98.2%, respectively (Figure 7F), and POD activity increased by 125.8% and 143.7%, respectively (Figure 7G). CAT activity increased by 31.9% under 2 mg/L AgNPs treatment (Figure 7H). Regardless of whether rice was exposed to AgNPs at the seed germination or seedling stage, APX activity in the rice leaves does not show significant differences compared to the control (Figure S7A,C). 

In addition to antioxidant enzymes, glutathione (GSH) is an antioxidant that protects plants from oxidative stress [41]. Exposure to a high concentration of AgNPs during the seed germination stage increased GSH content. Specifically, the GSH content was significantly increased by 32.7% compared to the control when exposed to 2 mg/L AgNPs (Figure S7B). Interestingly, no significant differences in GSH content were observed with AgNPs exposure during the seedling stage (Figure S7D). 

In summary, AgNPs exposure impacts antioxidant enzyme activities in rice leaves and varies depending on the growth stage. At the seed germination stage, AgNPs exposure has minimal effect on antioxidant enzyme activities in the rice leaves. Conversely, at the seedling stage, increasing AgNPs concentrations lead to a gradual increase in the activities of SOD, POD, and CAT in the rice leaves to eliminate excessive oxidative stress.

### 2.7. Effects of AgNPs on Protein Content and Antioxidant Enzyme Activity in Rice Roots

**Figure 8:**
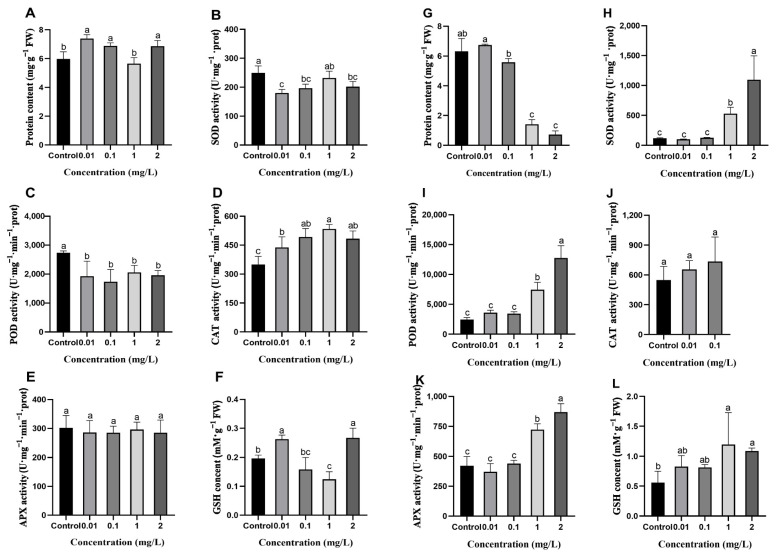
Effects of AgNPs exposure at seed germination and seedling stages on antioxidant enzyme activities in rice roots. AgNPs exposure at the seed germination stage: (**A**) protein content, (**B**) SOD activity, (**C**) POD activity, (**D**) CAT activity, (**E**) APX activity, and (**F**) GSH content. AgNPs exposure at the seedling stage: (**G**) protein content, (**H**) SOD activity, (**I**) POD activity, (**J**) CAT activity, (**K**) APX activity, and (**L**) GSH content. Data are mean ± SD. According to Tukey’s test (*p* < 0.05), different lowercase letters indicate significant differences among treatments, *n* ≥ 3.

Rice roots accumulate more silver elements compared to the shoots under AgNPs exposure, suggesting that roots may serve as the primary site of AgNPs accumulation. Therefore, this study further investigates the effects of AgNPs during the seed germination and seedling stage on the antioxidant enzyme activity in rice roots. The results showed that exposure to AgNPs at the seed germination stage significantly increased root protein content with the exception of the 1 mg/L AgNPs treatment (Figure 8A). However, under AgNPs exposure at the seedling stage, root protein content decreased with increasing AgNPs concentrations, with 1 and 2 mg/L AgNPs treatments resulting in a significant reduction of 77.7% and 88.6%, respectively, compared to the control (Figure 8G). 

Exposure of AgNPs during seed germination resulted in a decrease in SOD and POD activities in the roots compared to the control (Figure 8B,C). CAT activity was significantly higher than in the control, with the highest CAT activity observed at 1 mg/L AgNPs treatment, showing a 53.0% increase compared to the control (Figure 8D). APX activity showed no significant difference with control (Figure 8E). Exposure to AgNPs at the seedling stage resulted in a gradual increase in root SOD, POD, and APX activities with increasing AgNPs concentrations. At low AgNPs concentrations (0.01 and 0.1 mg/L), SOD, POD, and APX activities showed no significant differences with the control. However, at high AgNPs concentrations (1 and 2 mg/L), SOD activity increased by 358.2% and 850.6% (Figure 8H), POD activity increased by 203.6% and 418.9% (Figure 8I), and APX activity increased by 71.1% and 106.4%, respectively, compared to the control (Figure 8K). In addition, CAT activity in the rice roots showed an increasing trend under low concentrations (0.01 and 0.1 mg/L) of AgNPs exposure, but there were no significant differences with the control (Figure 8J). At high concentrations (1 and 2 mg/L), CAT activity in the rice roots was undetected, possibly due to very low contents. 

Variations in GSH content in rice roots were observed following AgNPs treatment at seed germination and seedling stages. Upon exposure to AgNPs at the seed germination stage, GSH content in rice roots significantly increased by 33.7% and 25.9% at 0.01 and 2 mg/L AgNPs, respectively, compared to the control. However, GSH content significantly decreased by 19.5% at 0.1 mg/L AgNPs (Figure 8F). Following exposure to AgNPs at the seedling stage, GSH content in rice roots increased with a higher AgNPs concentration. There was no significant difference in GSH content between a low AgNPs concentration (0.01 and 0.1 mg/L) and control, but at high concentrations (1 and 2 mg/L), GSH content increased by 137.5% and 94.6%, respectively, compared to the control (Figure 8L). 

In summary, the impact of AgNPs on protein content and antioxidant enzyme activities in rice roots varies with the growth stage. At the seed germination stage, AgNPs exposure has a minimal effect. Conversely, at the seedling stage, increasing AgNPs concentrations lead to a gradual increase in the activities of SOD, POD, and CAT in the rice leaves, which helps in the elimination of excessive oxidative stress and maintain homeostasis.

### 2.8. Effects of AgNPs on Nutritional Elements in Rice Shoots

Exposure to high concentrations of AgNPs at the seed germination stage did not affect Na, K, or Fe in the shoots of continuously growing rice (Table 1). However, at the seedling stage, high AgNPs concentrations significantly reduced the Na, K, and Fe contents in the shoots. AgNPs exposure at the seed germination stage increased the Mg content significantly in rice shoots. However, at the seedling stage, Mg content significantly decreased under AgNPs exposure. Specifically, 1 and 2 mg/L AgNPs treatments reduced the Mg content by 62.7% and 57.7%, respectively, compared to the control. The P content in rice shoots remained unchanged, regardless of AgNPs exposure at the seed germination or seedling stage.

## 3. Discussion

### 3.1. Rice Roots Are the Primary Accumulation Site for AgNPs

In this study, rice accumulates Ag in both roots and shoots during the seed germination and seedling stages under AgNPs exposure (Figure 1). Studies have shown that AgNPs can enter through the root epidermis [42], be absorbed by the root cap, accumulate in the columella cells [43], or be translocated within the plant to other tissues [43,44]. Quah et al. [45] research also show that under AgNPs stress, both the roots and shoots of *Glycine max* and *Triticum aestivum* accumulate Ag. Our study reveals that when rice is exposed to silver nanoparticles during the seed germination and seedling stages, the roots are the main site of silver accumulation, with significantly higher accumulation in the roots than in the leaves (Figure 1). Specifically, at the seedling stage, roots accumulated 34 to 39 times more Ag than shoots at concentrations of 1 and 2 mg/L of AgNPs, demonstrating a notable concentration-dependent accumulation. This finding aligns with the previous findings that roots are the principal sites and a major sink for the uptake and retention of AgNPs due to their direct interaction with nanoparticles in the growth medium [23,25,42,43,44,46,47]. 

The exposure of AgNPs during the seed germination stage resulted in lower Ag accumulation in coleoptiles and radicles than in shoots and roots at the seedling stage (Figure S1 vs. Figure 1). This difference is likely due to the protective role of the seed coat, which acts as a barrier to AgNPs absorption [38]. Alternatively, the selective permeability of the seed coat during germination may reduce the sensitivity of seeds to different concentrations of AgNPs [48], leading to decreased AgNPs absorption at the seed germination stage. At the seedling stage, the absence of this barrier, combined with the higher surface area of roots and their active uptake mechanisms, facilitated higher Ag accumulation in both shoots and roots. Interestingly, Ag content decreased in seedlings exposed to AgNPs during germination as their biomass increased (Figure 1A,B), suggesting that increased biomass reduces the concentration of accumulated Ag, potentially minimizing its impact on growth. In contrast, AgNPs exposure at the seedling stage resulted in much higher accumulation, particularly in roots, and was associated with growth inhibition at higher concentrations. This stage-dependent accumulation pattern highlights the role of developmental factors in determining plant susceptibility to nanoparticles.

### 3.2. Differential Effects of AgNPs on Rice Growth and Development at Seed Germination and Seedling Stages

The results of this study indicate that rice responds differently to AgNPs exposure depending on the developmental stage. AgNPs exposure during the seed germination stage did not significantly affect the later growth of rice seedlings, even at higher concentrations. This observation is consistent with our findings of lower Ag accumulation in coleoptiles and radicles during germination, which can be attributed to the protective role of the seed coat, limiting AgNPs absorption (Figure S1). The accumulation of Ag in rice shoots and roots treated with 2 mg/L AgNPs at the seed germination stage was lower than that in rice treated with 0.1 mg/L AgNPs at the seedling stage (Figure 1). Similarly, Stampoulis et al. [49] reported that even at a high concentration of 1000 mg/L, AgNPs suspension did not hinder the germination and growth of *Cucurbita pepo*. In contrast, exposure to AgNPs during the seedling stage significantly inhibited growth and biomass accumulation in rice, with effects intensifying at higher concentrations. Compared to the seed germination process, the root development of rice seedlings is more advanced and capable of absorbing more substances [50]. Ke et al. [37] observed a more pronounced inhibition of *Arabidopsis* fresh weight with increased concentrations of AgNPs. Consequently, these effects can be linked to the substantially higher Ag accumulation in roots during the seedling stage, where roots directly interact with AgNPs in the growth medium, thereby inhibiting plant growth and development.

Furthermore, exposure to AgNPs during the seedling stage disrupted nutrient balance in rice leaves, as evidenced by the decrease in Na, K, Fe, and Mg levels. This disruption is consistent with previous studies reporting that nanoparticle exposure can impair ion absorption and accumulation [51,52,53,54,55]. External intake of nutritional elements is essential for the growth and development of plants [51]. The ion transport disruption caused by AgNPs likely diminishes the availability of essential nutrients [55]. Moreover, the penetration of Ag into cells can harm cell membranes and disrupt ion balance within the cell [56]. As a result, exposure to AgNPs during the seedling stage may lead to increased Ag uptake by rice roots, diminishing their nutrient absorption capacity, impacting rice growth and development, and significantly reducing biomass.

### 3.3. Effects of AgNPs on the Antioxidant System and Photosynthetic Efficiency of Rice at Seed Germination and Seedling Stages 

Abiotic stress produces ROS and free radicals in plants, causing oxidative damage [57]. To combat this, plants have developed a defense system that boosts antioxidant enzyme activities or accumulates antioxidant compounds to eliminate ROS and free radicals [58,59,60,61,62]. The results demonstrate oxidative stress in response to AgNPs and this response differed depending on the developmental stage. At the seed germination stage, exposure to AgNPs resulted in no significant oxidative damage in rice tissues during later growth. Specifically, the levels of ROS in rice leaves were reduced compared to the control group (Figure S6A), while MDA content in both leaves and roots remained comparable to the control (Figure 5B and Figure 6A). Additionally, 3,3′-diaminobenzidine (DAB) staining indicated that H_2_O_2_ levels in the leaves showed no significant differences from the control group (Figure 5A). These findings suggest that AgNPs exposure during the seed germination stage does not induce substantial oxidative stress in rice. This is likely due to the lower accumulation of Ag in the rice tissues at the germination stage (Figure 1A,B), with the seed coat acting as a protective barrier that restricts AgNPs penetration. This observation aligns with previous reports where seeds exhibited lower sensitivity to nanomaterials due to the selective permeability of the seed coat or metabolic quiescence during germination [55,63]. Moreover, there were no significant variations in the levels of antioxidant enzymes, such as SOD, POD, and APX, in both rice leaves and roots when compared to the control (Figure 7B,C and Figure S7A). This aligns with the observed levels of oxidative damage in the rice tissues, supporting the notion that the oxidative stress response at the germination stage is minimal and consistent with the lower Ag accumulation in rice tissues [55]. 

In contrast, AgNPs exposure at the seedling stage resulted in substantial oxidative damage. DAB staining results revealed that H_2_O_2_ content in the leaves increased with higher concentrations of AgNPs (Figure 5C). Nair and Chung [27] also obtained similar results from the toxicity of AgNPs in rice shoots. Increasing the H_2_O_2_ content in rice following AgNPs stress is due to the transportation of AgNPs from root to shoot as indicated by Ag content [64]. ROS levels in the roots gradually increased with higher concentrations of AgNPs (Figure 6D). This could be attributed to the direct contact of the rice roots with the AgNPs solution, with the roots serving as the primary target for AgNPs and absorbing significant amounts of Ag elements. As a result, the roots of the seedlings are likely to experience more significant oxidative damage than the leaves due to a higher accumulation of Ag in the roots. These findings align with those of Huang et al. [25], who showed that roots are the primary target of AgNPs uptake, with limited translocation to shoots due to extracellular barriers. Additionally, as the AgNPs concentration increases, the activities of antioxidant enzymes such as SOD, POD, and CAT in rice leaves gradually rise (Figure 7F–H). Similarly, antioxidant enzymes, including SOD, POD, and APX, also exhibit a concentration-dependent increase in rice roots with AgNPs exposure (Figure 8H,I,K). These results are consistent with previous findings showing higher activities of antioxidant enzymes under AgNPs exposure [44,61,62,65]. This study also observed that exposure to high concentrations of AgNPs led to the inability to measure the activity of the CAT enzyme in rice roots (Figure 8J). This could be attributed to the non-specific degradation of the CAT protein or its inactivation due to excessive free radical production [66]. 

Photosynthesis is a fundamental metabolic process in green plants and is the primary source of ROS production in their cells [67]. AgNPs-induced oxidative stress in plants is closely associated with photosynthesis [68]. Chlorophyll fluorescence parameters can indicate plants’ health status and tolerance to stressful environments [69,70]. The OJIP fluorescence transient curve reflects changes in the primary photochemical reactions of PSII and the status of the photosynthetic apparatus [71]. Previous studies reported that exposure to 1 mg/L AgNPs affects the OJIP transient fluorescence curve of Lemnaceae (*Ottelia alismoides*) fronds, causing damage to the photosynthetic apparatus [72]. In the present study, OJIP fluorescence transient curves results demonstrated that exposure to AgNPs at the seed germination stage has minimal impact on the PSII of rice seedlings during later growth (Figure 4B). However, high concentrations of AgNPs at the seedling stage affected PSII electron transport and the conversion of light energy to chemical energy in rice seedlings during later growth (Figure 4D). This suggests that exposure to AgNPs during the seedling stage of rice affects the photosynthetic capacity to some extent. Photosynthesis is crucial for plant growth and development [73], and the reduced photosynthetic capacity in rice seedlings after AgNPs stress is one of the reasons for its diminished growth and development.

## 4. Materials and Methods

### 4.1. Plant Materials and AgNPs Treatment

The study utilized the indica rice (*Oryza sativa* L.) cultivar Huanghuazhan, provided by the Photosynthetic Stress Research Center at South China Agricultural University. Polyvinylpyrrolidone-coated silver nanoparticles (PVP-AgNPs), procured from NanoComposix (San Diego, CA, USA), featured a concentration of 4500 mg/L and a particle diameter of 5 nm. The AgNPs solutions were prepared at concentrations of 0 (control), 0.01, 0.1, 1, and 2 mg/L for experimental treatments.

### 4.2. Plant Growth and Experimental Design

AgNPs exposure during the rice seed germination stage. Rice seeds were sterilized in a 0.1% H_2_O_2_ solution for 6 h, followed by thorough rinsing with distilled water. Subsequently, the seeds were immersed in AgNPs solutions at concentrations of 0 (control), 0.01, 0.1, 1, and 2 mg/L concentrations. Seeds immersed in deionized water served as the control. Each treatment included 100 seeds. After a 42-h soaking period at 30 °C in a growth chamber, the seeds were placed in Petri dishes lined with filter paper and incubated for germination. During the germination period, AgNPs solutions of the respective concentrations were applied to maintain moisture levels. Germination was considered to have occurred when the radical emerged from the seed coat. After 5 days of exposure to AgNPs during the seed germination, half of the seedlings were collected for Ag content determination. The remaining seedlings were transferred to 96-well culture boxes containing Kimura B nutrient solution and grown for 13 days under controlled conditions (25 ± 1 °C, 16:8 h light: dark photoperiod, and 160 μmol photon m^−2^ s^−1^ light intensity). Growth and physiological indices were subsequently assessed.

AgNPs exposure during the seedling stage. Rice seeds were sterilized in a 0.1% H_2_O_2_ solution for 6 h, rinsed thoroughly, and soaked in distilled water for 42 h at 30 °C. After this period, the seeds were transferred to Petri dishes lined with filter paper and incubated for 48 h to germinate. Germination was considered to have occurred when the radical emerged from the seed coat. The germinated seeds were then transferred to 96-well culture boxes and grown in a greenhouse for 4 days. Uniformly grown rice seedlings were selected, wrapped in foam strips, and secured in 6-well hydroponic culture boxes (2 seedlings per well). The seedlings were grown for 5 days in Kimura B nutrient solution. Subsequently, AgNPs solutions of 0 (control), 0.01, 0.1, 1, and 2 mg/L concentrations were added to the nutrient solution hydroponic treatment. After 5 days of AgNPs exposure, the growth and physiological indices were measured. 

### 4.3. Nutrient Elements and Ag Accumulation Measurement in Rice Tissues

The rice seedlings were separated into shoots and roots. The roots were washed thrice with 10 mM EDTA-Na_2_ solution and then rinsed with ultrapure water. The shoots and roots were dried at 60 °C in an oven until a constant weight was achieved and ground into powder using a mortar and pestle. Each sample was then weighed to 20 mg and digested using a microwave digestion system with 2 mL of concentrated nitric acid. After cooling, the samples were degassed by ultrasound for 5 min, diluted to 25 mL with ultrapure water, and the nutrient elements in the shoot and root samples were determined using ICP-OES (Plasma 3000, Agilent Technologies, Santa Clara, CA, USA). The Ag content in the shoot and root samples was determined using ICP-MS (NexlON 5000G, PerkinElmer, Shelton, CT, USA).

### 4.4. Estimation of Photosynthetic Pigments

Fresh leaves (0.01 g) were extracted in 2 mL of 95% ethanol and left at 4 °C in the dark for 24 h to extract pigments. A UV–Vis spectrophotometer (T6, Beijing Purkinje GENERAL Instrument Co., Ltd., Beijing, China) was used to measure the absorbance at wavelengths of 665 nm, 649 nm, and 470 nm. The pigment content, including chlorophyll a (Chl a), chlorophyll b (Chl b), carotenoids (Car), and total chlorophyll (Tchl), were calculated according to Formulas (1)–(4) [74]. The results were expressed as the pigment content per gram of leaf (fresh weight, FW).

Chl a (mg/L) = 13.95 × A665 − 6.88 × A649(1)

Chl b (mg/L) = 24.96 × A649 − 7.32 × A665(2)

Car (mg/L) = (1000 × A470 − 2.05 × Chl a − 114.8 × Chl b)/245(3)

Tchl = Chl a + Chl b(4)

### 4.5. Estimation of Chlorophyll Fluorescence Parameters

Chlorophyll fluorescence parameters were obtained by a portable chlorophyll fluorometer PAM2500 (Walz, Heinz Walz GmbH, Effeltrich, Germany). After 15–30 min dark adaptation, the F_v_/F_m_, the rETR_max_, and the OJIP fluorescence transient curves were recorded. The OJIP data were standardized by normalizing the raw data with the fluorescence intensity Ft at point O to 0. Each treatment had at least 6 biological replicates.

### 4.6. Estimation of Oxidative Stress in Leaves and Roots

The rice leaves were stained with DAB to visualize the H_2_O_2_ content as the method described by Thordal-Christensen et al. [75]. Rice leaves from the same position were cut and immersed in the DAB staining solution. Afterward, they were transferred to a growth chamber with a light intensity of 500 µmol m^−2^ s^−1^ for 1 h, submerged in 95% ethanol, and heated in a 90 °C water bath to remove chlorophyll. The MDA content was determined with some modifications based on the thiobarbituric acid (TBA) method described by Heath and Packer [76]. The ROS was determined with fluorescence intensity following the method described by Oukarroum et al. [77]. The fluorescence intensity data were normalized by fresh weight to compare the differences in ROS content between different treatments.

### 4.7. Estimation of Protein Content and Antioxidant Enzymes

Fresh root (0.2 g) and leaf (0.05 g) tissues were homogenized in a phosphate buffer solution (0.05 M, pH 7.8). After centrifugation at 6000 rpm and 4 °C for 10 min. The supernatant was collected to determine the protein content and antioxidant system enzyme activities. The protein content was determined using the method described by Bradford [78]. SOD activity was determined using the nitroblue tetrazolium (NBT) colorimetric method described by Beauchamp and Fridovich [79]. POD activity was determined with some modifications based on the guaiacol method described by Herzog and Fahimi [80]. CAT activity was determined using the hydrogen peroxide consumption method described by Damanik et al. [81]. APX activity was determined with some modifications based on the ascorbate oxidation method described by Nakano and Asada [82]. GSH content was determined with some modifications based on the method described by Griffith et al. [83].

### 4.8. Data Analysis

All the data are expressed as mean and standard deviation. One-way ANOVA was performed to test the significant differences between treatments, followed by the Tukey test when *p* < 0.05 was statistically significant (spss 23.0). Graphical representations of the data were generated using GraphPad Prism 8 software.

## 5. Conclusions

This study found that rice at seed germination and seedling stages exhibits distinct absorption and stress response mechanisms to AgNPs. Rice seedlings exposed to AgNPs accumulate more Ag than those exposed during the seed germination stage. This may be due to the hard seed coat, which partially blocks the entry of AgNPs. Furthermore, as the rice grows and its biomass increases, the concentration of Ag in the plant decreases. Therefore, exposure to AgNPs during the seed germination stage does not inhibit later growth, nor does it cause significant damage to the photosynthetic system or induce oxidative stress. In contrast, during the seedling stage, the more developed root system allows for more efficient absorption of AgNPs, leading to a dose-dependent increase in Ag accumulation. This accumulation inhibits seedling growth, damages the photosynthetic system, and induces oxidative stress. As the concentration of AgNPs increases, the H_2_O_2_ content in the leaves rises, and the activity of antioxidant enzymes (SOD, POD, CAT) also increases in a dose-dependent manner. Additionally, the roots of rice are the primary site for Ag absorption and accumulation, making the roots more susceptible to oxidative damage from AgNPs than the leaves.

## Figures and Tables

**Table 1:** Effects of AgNPs exposure at the seed germination and seedling stages on nutritional elements in rice seedling shoots.

	Nutrient (μg/g DW)	Control	0.01 mg/L	0.1 mg/L	1 mg/L	2 mg/L
Seed germination stage	Fe	39,627.9 ± 1547.9 ^b^	46,450.0 ± 1072.2 ^a^	49,024.5 ± 391.7 ^a^	47,873.7 ± 1843.0 ^a^	47,999.5 ± 2547.6 ^a^
	K	3099.2 ± 128.2 ^a^	3766.0 ± 386.7 ^a^	3936.0 ± 351.6 ^a^	3802.4 ± 580.8 ^a^	3824.7 ± 348.8 ^a^
	Mg	724.0 ± 46.6 ^b^	1198.4 ± 72.6 ^a^	1243.3 ± 88.2 ^a^	1255.6 ± 136.5 ^a^	1049.2 ± 100.2 ^a^
	Na	12,012.9 ± 248.3 ^b^	11,754.8 ± 138.3 ^b^	13,285.2 ± 196.4 ^a^	12,652.0 ± 952.9 ^ab^	11,773.8 ± 360.0 ^b^
	P	2450.7 ± 64.3 ^a^	2168.2 ± 136.8 ^a^	1850.6 ± 280.1 ^a^	22,967.0 ± 468.9 ^a^	2160.5 ± 261.7 ^a^
Seedling stage	Fe	41,301.7 ± 1222.2 ^a^	38,474.6 ± 2069.0 ^a^	38,687.7 ± 1948.0 ^a^	28,298.2 ± 3509.6 ^b^	28,224.5 ± 1370.7 ^b^
	K	3835.9 ± 230.4 ^a^	3338.3 ± 717.5 a^b^	3111.4 ± 1009.0 ^ab^	869.6 ± 1229.8 ^c^	1865.5 ± 413.4 ^bc^
	Mg	2217.6 ± 18.4 ^a^	1937.2 ± 101.7 ^b^	1831.1 ± 71.9 ^b^	827.2 ± 123.7 ^c^	937.4 ± 41.9 ^c^
	Na	12,983.2 ± 359.9 ^a^	12,262.8 ± 375.4 ^a^	13,053.9 ± 564.8 ^a^	9694.7 ± 560.7 ^b^	8121.3 ± 191.8 ^c^
	P	2393.6 ± 368.1 ^a^	2576.6 ± 803.8 ^a^	2833.3 ± 1217.4 ^a^	3550.7 ± 1374.1 ^a^	1996.1 ± 281.8 ^a^

Data are mean ± SD. According to Tukey’s test (*p* < 0.05), different lowercase letters indicate significant differences among treatments, *n* ≥ 3.

## Data Availability

Data are contained within the article. Further inquiries can be directed to the corresponding authors.

## Supplementary Materials

The following supporting information can be downloaded at https://www.mdpi.com/article/10.3390/plants13233454/s1: Figure S1: Ag content in (A) coleoptile, (B) radicle, and (C) endosperm after 5 days of AgNPs exposure during seed germination; Figure S2: Effects of AgNPs exposure at seed germination and seedling stages on rice growth parameters; Figure S3: Leaf pigment content exposure to AgNPs at the seed germination stage; Figure S4: Leaf pigment content exposure to AgNPs at the seedling stage; Figure S5: Effects of AgNPs exposure at seed germination and seedling stages on F_v_/F_m_ in rice leaves; Figure S6: Effects of AgNPs exposure at seed germination and seedling stages on ROS in rice leaves; Figure S7: Effects of AgNPs exposure at seed germination and seedling stages on antioxidant enzyme activities in rice seedling leaves.
