# Supplementary material for: Stage-Specific Effects of Silver Nanoparticles on Physiology During the Early Growth Stages of Rice
Source: Plants (Basel). 2024 Dec 9;13(23):3454. doi: 10.3390/plants13233454 (PMC11644278; doi:10.3390/plants13233454)
Supplement: Supplementary file 1 [file plants-13-03454-s001.zip › plants-3314947-supplementary.pdf]

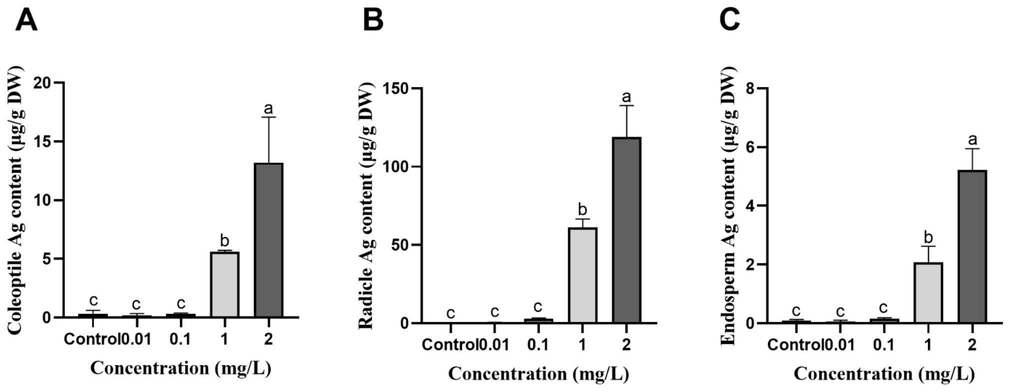

Figure S1. Ag content in (A) coleoptile, (B) radicle, and (C) endosperm after 5 days of AgNPs exposure during seed germination. Data are mean ± SD. According to Tukey´s test ($p < 0.05$), different lowercase letters indicate significant differences among treatments, n = 3.

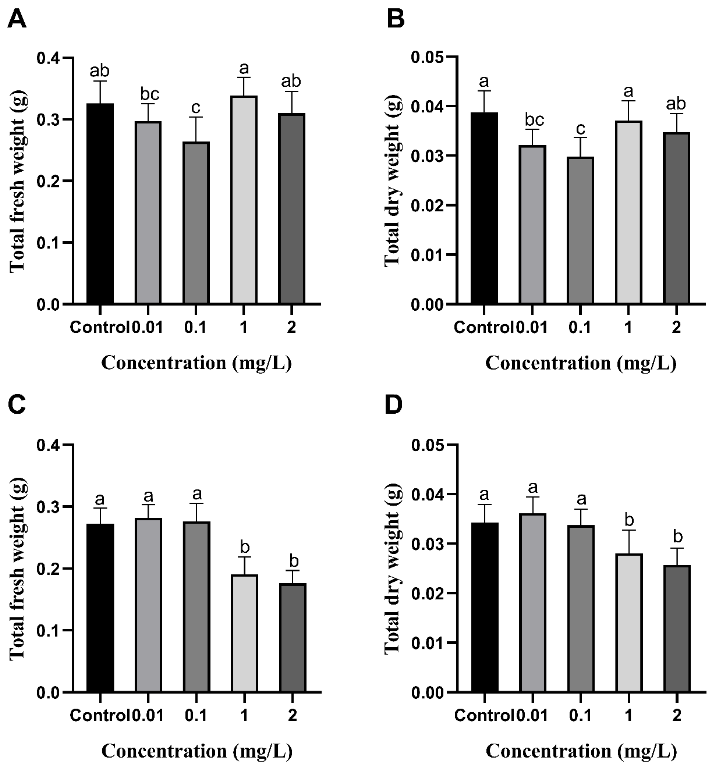

Figure S2: Effects of AgNPs exposure at seed germination and seedling stages on rice growth parameters. AgNPs exposure at the seed germination stage. (A) total fresh weight; (B) total dry weight. AgNPs exposure at the seedling stage: (C) total fresh weight; (D) total dry weight. Data are mean ± SD. According to Tukey´s test ($p < 0.05$), different lowercase letters indicate significant differences among treatments, n ≥ 4. In the seed germination stage treatment, rice seeds were soaked in AgNPs for 5 days, then the rice seedlings were grown normally in Kimura B nutrient solution for 18 days. In the seedling stage treatment, after normal germination and growth of rice for 18 days, 5 days of Ag exposure.

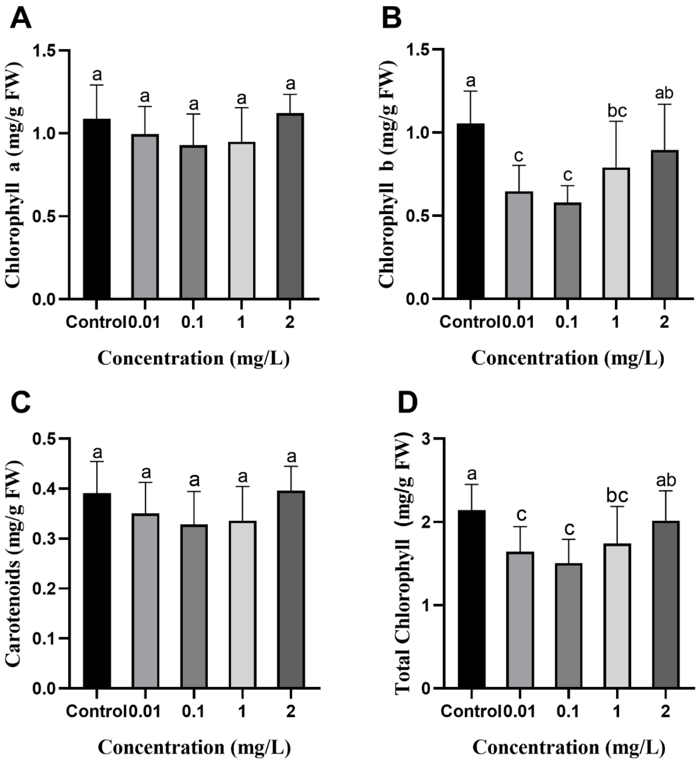

Figure S3: Leaf pigment content eposure to AgNPs at the seed germination stage. (A) Chlorophyll a, (B) Chlorophyll b, (C) Carotenoids content , and (D) Total Chlorophyll.. Data are mean ± SD. According to Tukey´s test ($p < 0.05$), different lowercase letters indicate significant differences among treatments, n ≥ 4.

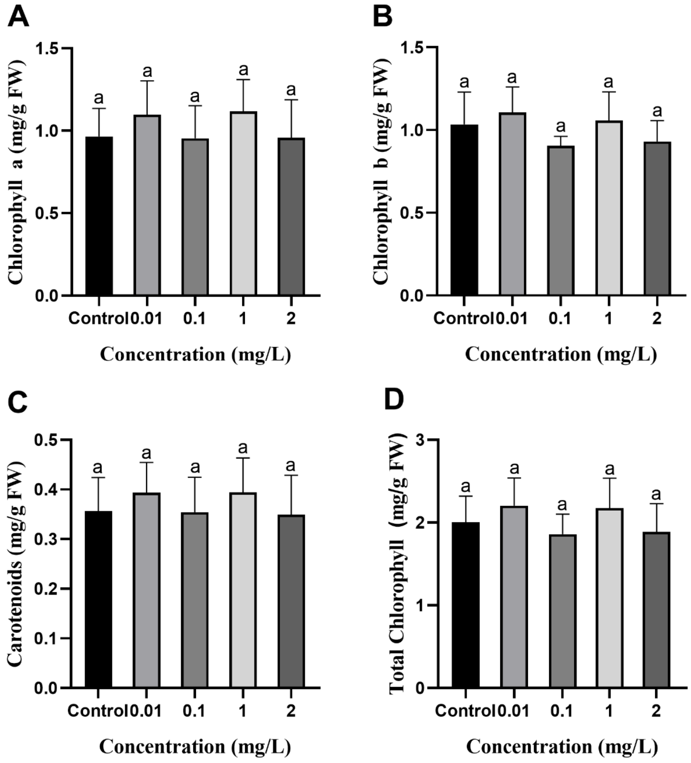

Figure S4: Leaf pigment content eposure to AgNPs at the seedling stage. (A) Chlorophyll a, (B) Chlorophyll b, (C) Carotenoids content , and (D) Total Chlorophyll.. Data are mean ± SD. According to Tukey´s test ($p < 0.05$), different lowercase letters indicate significant differences among treatments, n ≥ 4.

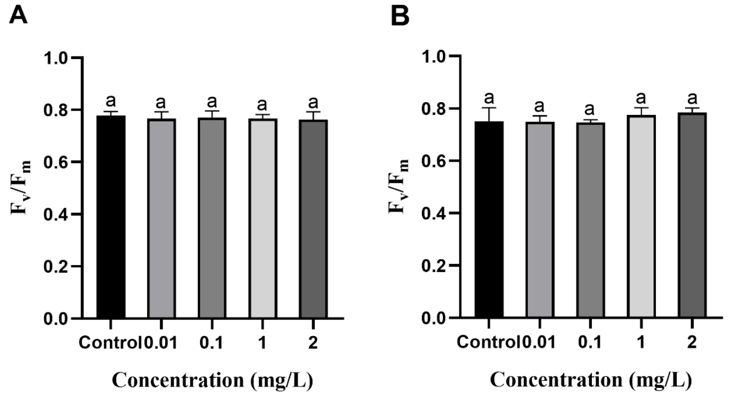

Figure S5: Effects of AgNPs exposure at seed germination and seedling stages on maximal quantum yield (F_v/F_m) in rice leaves. (A) Rice leaves F_v/F_m under AgNPs exposure at the seed germination stage; (B) Rice leaves Fv/Fm under AgNPs exposure at the seed germination stage. Data are mean ± SD. According to Tukey´s test ($p < 0.05$), different lowercase letters indicate significant differences among treatments, n ≥ 4.

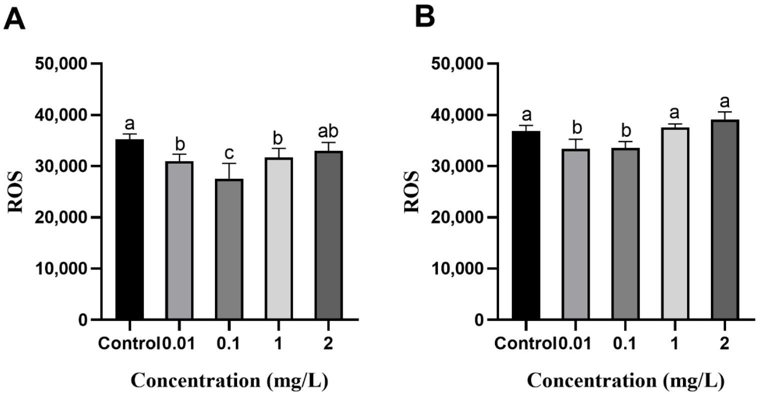

Figure S6: Effects of AgNPs exposure at seed germination and seedling stages on reactive oxygen species (ROS) in rice leaves. (A) ROS of rice leaves under AgNPs exposure at the seed germination stage; (B) ROS of rice leaves under AgNPs exposure at the seed germination stage. Data are mean ± SD. According to Tukey´s test ($p < 0.05$), different lowercase letters indicate significant differences among treatments, n ≥ 4.

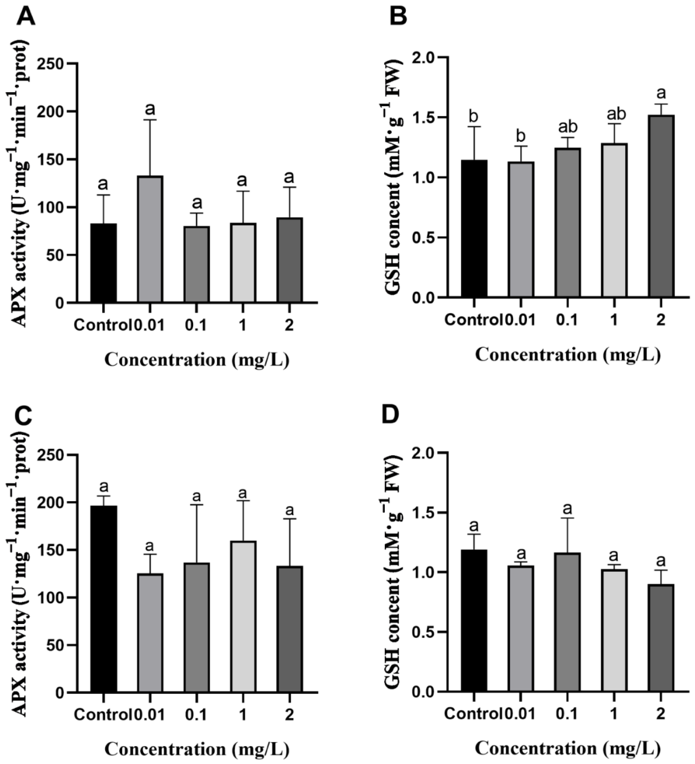

Figure S7. Effects of AgNPs exposure at seed germination and seedling stages on antioxidant enzyme activities in rice seedling leaves. AgNPs exposure at the seed germination stage: (A) ascorbate peroxidase (APX) activity, and (B) glutathione (GSH) concent. AgNPs exposure at the seedling stage: (C) APX activity, and (D) GSH concent. Data are mean ± SD. According to Tukey′s test ($p < 0.05$), different lowercase letters indicate significant differences among treatments, $n \geq 4$.
